# Functional implications of multiseriate cortical sclerenchyma for soil resource capture and crop improvement

**DOI:** 10.1093/aobpla/plac050

**Published:** 2022-10-18

**Authors:** Hannah M Schneider

**Affiliations:** Centre for Crop Systems Analysis, Wageningen University & Research, Wageningen 6708 PE, The Netherlands

**Keywords:** Anatomy, cortex, edaphic stress, multiseriate cortical sclerenchyma, root

## Abstract

Suboptimal nutrient and water availability are primary constraints to crop growth. Global agriculture requires crops with greater nutrient and water efficiency. Multiseriate cortical sclerenchyma (MCS), a root anatomical trait characterized by small cells with thick cell walls encrusted with lignin in the outer cortex, has been shown to be an important trait for adaptation in maize and wheat in mechanically impeded soils. However, MCS has the potential to improve edaphic stress tolerance in a number of different crop taxa and in a number of different environments. This review explores the functional implications of MCS as an adaptive trait for water and nutrient acquisition and discusses future research perspectives on this trait for incorporation into crop breeding programs. For example, MCS may influence water and nutrient uptake, resistance to pests, symbiotic interactions, microbial interactions in the rhizosphere and soil carbon deposition. Root anatomical phenotypes are underutilized; however, important breeding targets for the development of efficient, productive and resilient crops urgently needed in global agriculture.

## Teaser

Root traits play a primary role in plant adaption to stress. Multiseriate cortical sclerenchyma (MCS) is an anatomical trait characterized by small cells encrusted with lignin in the outer cortex. Progress has recently been achieved in understanding the development and physiological implications of MCS. This review proposes that MCS is a useful trait for water and nutrient acquisition, particularly in edaphic stress conditions. Multiseriate cortical sclerenchyma may be a useful breeding target for improved soil resource capture and biotic stress tolerance in several major crop species including wheat, maize, sorghum and barley.

## Introduction

Sustainable crop production is a major challenge for humanity as the global population and food demand continue to grow. Humanity urgently requires crops with greater water and nutrient efficiency that are more productive in degraded soils with fewer fertilizer, water and chemical inputs. To meet the demands of the growing population, food production will need to increase by 25–50 % in the next 30 years (Hunter *et al.* 2017). Climate change adds further complexity to this challenge as altered precipitation patterns, heat stress, soil degradation and depletion of freshwater resources will reduce the regions and number and length of growing seasons suited for crop production (IPCC 2021). One of the greatest challenges facing humanity is to produce higher crop yields in the face of climate change with fewer inputs (Anderson *et al.* 2020).

One pragmatic solution for addressing these grand challenges lies in the breeding of improved crop cultivars with enhanced resource use efficiency. Although phenotypic variation in above-ground tissues can have important impacts on water and nutrient utilization efficiency in crops, roots are the primary organ for nutrient and water uptake and are the interface of plants with soils (Fageria *et al.* 2008). Variation in root phenotypes can have significant effects on soil resource acquisition by modifying the placement of roots in the soil where limiting resources are most available, improving the metabolic efficiency of soil foraging, influencing the radial and axial transport of resources and altering the mechanical strength of the root (Lynch 2019; Lynch *et al.* 2021b; Amtmann *et al.* 2022).

The main purpose of this review is to highlight multiseriate cortical sclerenchyma (MCS) as a crop breeding target and draw attention to open questions and the potential of this trait for improved plant stress tolerance and, ultimately, plant performance and yield. This review will not aim to provide a comprehensive review of MCS; however, it will focus on opportunities and perspectives for studying this trait and its potential for application in crop breeding programs.

## MCS Is an Important Trait for Stress Tolerance

**Figure 1.**
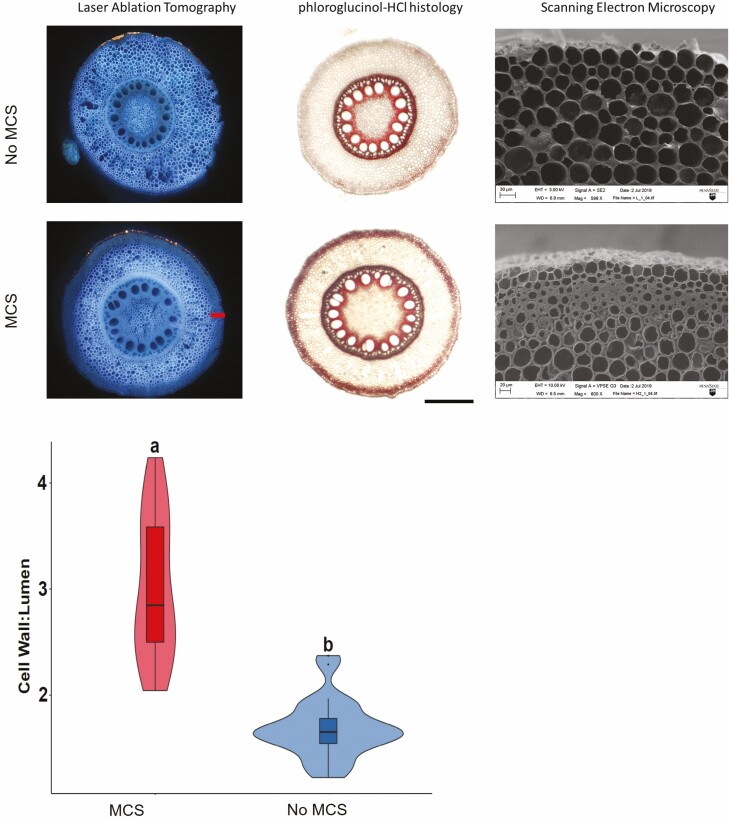
Lines with MCS have smaller and thicker outer cortical cells. These thickened cortical cells are stained with phloroglucinol–HCl due to their high lignin content. Cryo-SEM images show detailed images of the smaller cells with thick cell walls in the outer cortex (scale bar, 100 µm). Violin plot showing median, interquartile range, 95 % CIs and frequency of cell wall:lumen area ratio for MCS (*n* = 27) and non-MCS (*n* = 26) root samples. Letters denote significant differences as determined by Welch’s two-sample *t*-test at a confidence level of *α* ≤ 0.05. Reprinted with permission from Schneider *et al.* (2021).

**Figure 2.**
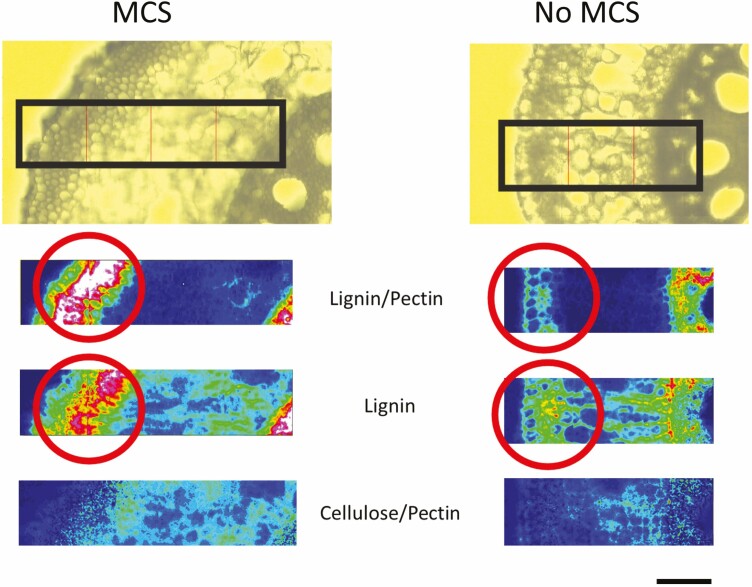
Fourier-transform infrared spectroscopy demonstrates that outer cortical cells in genotypes with MCS are embedded with lignin (highlighted in red circles) and do not have elevated concentrations of pectin or cellulose when compared to non-MCS genotypes (*n* = 2). The heatmap displays the concentration of the cell wall component and the hotter the colour, the greater the concentration. Images are of nodal, field-grown maize roots (scale bar, 50 µm). Reprinted with permission from Schneider *et al.* (2021).

Multiseriate cortical sclerenchyma is characterized by small sclerenchymatous cells with thick cell walls encrusted with lignin in the outer cortex of nodal roots in several *Poaceae* species including maize, wheat, barley and sorghum (Fig. 1). Developmentally, MCS forms in axial roots, primarily in younger nodal roots and persists throughout plant maturity. Multiseriate cortical sclerenchyma has not been observed in seminal or lateral roots. In maize, the MCS phenotype was observed after the zone of differentiation and lateral root formation in axial nodal roots. Unlike other anatomical traits, cortical cells with MCS do not change their cell wall thickness once MCS has formed (Schneider *et al.* 2021; Clément *et al.* 2022). Roots with MCS have significantly more lignin in the root (Fig. 2) and root tip compared to roots without MCS. Experiments have also demonstrated that MCS development is modulated by ethylene exposure and has a relatively high heritability (Schneider *et al.* 2021). Multiseriate cortical sclerenchyma can be quantitatively phenotyped through measuring the cell wall-to-lumen ratio in outer cortical cells. Maize, wheat and barley lines with a cell wall-to-lumen area ratio of greater than two are classified as having MCS (Fig. 1) (Schneider *et al.* 2021).

The MCS phenotype is present in some modern maize inbreds but not in accessions of teosinte *parviglumis* and *Z. mays* ssp. *mexicana* (teosinte *mexicana*) suggesting it might represent an adaptation acquired during domestication (Schneider *et al.* 2021). Multiseriate cortical sclerenchyma also has not been observed in landraces of wheat or barley, but is present in many modern wheat and barley cultivars (Schneider *et al.* 2021). It has been proposed that crop domestication included selection for adaption to more challenging soil environments (Lynch *et al.* 2021a). However, interestingly MCS is not found in the wild ancestors of cereal crops (e.g. teosinte), but is present in many cultivated lines, including in 5280-year-old maize roots from Mexico (Lopez-valdivia *et al.* 2022).

The composition and size of cortical cells have been shown to be important for soil penetration in hard soils (Chimungu *et al.* 2015; Colombi and Walter 2016; Huang *et al.* 2022). Several physical soil properties such as water content, texture and bulk density influence mechanical impedance (Gao *et al.* 2016; Vanhees *et al.* 2020) and can limit root elongation, crop growth and, subsequently, crop yield (Whalley *et al.* 2005; Whitmore and Whalley 2009; Bengough *et al.* 2011; Gao *et al.* 2016). Sclerenchymatous tissue is generally characterized by complexes of thick cell walls encrusted with lignin that enable plant organs to withstand stretching, bending and pressure strains (Evert 2006). Multiseriate cortical sclerenchyma encrusted with lignin enhanced the tensile strength of the root by 24 % and the bending force of the root tip by 109 % which enabled increased penetration in mechanically impeded soils (Schneider *et al.* 2021). In the field, maize genotypes with MCS had root systems with 22 % greater depth and 49 % greater shoot biomass in compacted soils compared to genotypes without MCS (Schneider *et al.* 2021). Similarly, smaller outer cortical cells in maize enhanced root tensile strength and root bending force (Chimungu *et al.* 2015) and plant growth in dry, hard soils (Klein *et al.* 2020). Multiseriate cortical sclerenchyma has an important role in the mechanical strength of the root for continued elongation into hard or dense soils. Soil cultivation in traditional agriculture generally leads to soil degradation through accelerated erosion, and loss of organic matter, biopores and soil structure which leads to increased mechanical impedance (Lynch *et al.* 2021a). Multiseriate cortical sclerenchyma is an important root trait that enables penetration of hard soils, typically found in modern agroecosystems.

## Future Perspectives and Research Opportunities for Understanding and Deploying MCS for Crop Improvement

There is great potential of MCS to be utilized in crop breeding programs for the development of more productive crops in a range of environments. Although MCS has been characterized as a trait that improves plant performance in mechanical impedance, many open research questions remain that may highlight this trait for adaptation to a number of different edaphic stresses. Here, several research gaps are presented that could be addressed in order to facilitate our understanding of MCS and its dynamic interactions with the plant and rhizosphere in many environments. Understanding the benefits and trade-offs of MCS in specific environments will facilitate the incorporation of this trait into crop breeding programs for the development of crops with edaphic stress tolerance and enhanced yield.

### MCS may influence the metabolic cost of soil exploration

Plants that are able to acquire soil resources at a reduced carbon and nutrient cost will have increased productivity due to greater allocation of resources to shoot and root growth, continued resource acquisition and reproduction (Lynch 2019). Different anatomical features require different investments and costs in metabolic resources and therefore are a primary determinant of the metabolic cost of root (Chimungu *et al.* 2014a; Colombi *et al.* 2019). For example, some types of sclerenchyma cells are dead and living cell types, including parenchyma, have varying proportions of polysaccharides, protein and nucleic acids. The proportion of living and dead cells, cell composition and cell activity are primary determinants of the metabolic costs of root construction and maintenance and, subsequently soil exploration (Lynch *et al.* 2021b).

Reducing the cortical burden and metabolic costs of soil exploration has been shown to be an adaptive trait and improves plant growth in many environments including drought (Zhu *et al.* 2010), suboptimal nitrogen availability (Saengwilai *et al.* 2014) and suboptimal phosphorus availability (Galindo-Castaneda *et al.* 2018). Several anatomical traits are important for reducing the metabolic cost of root foraging. For example, a greater cortical cell size (Chimungu *et al.* 2014b; Colombi *et al.* 2019) and fewer cortical cell files (Chimungu *et al.* 2014a) enhance plant growth in drought by reducing respiration and root construction and maintenance costs. Larger cells have proportionately greater vacuole/cytoplasm volume, as the cytoplasm is more metabolically active than the vacuole. In addition, by reducing the number of cortical cell files, the number of parenchyma cells in the cortex is reduced, which alters the metabolic cost of root construction and maintenance. Similarly, MCS may impact the metabolic costs for root construction and maintenance through modifications in cell size (Chimungu *et al.* 2014b; Colombi *et al.* 2019), cell wall thickness and cell wall composition. It has been proposed that the lignified cell walls in MCS are less metabolically active than cortical parenchyma. If roots with MCS have a reduced metabolic cost of root construction and/or maintenance, this may allow for a greater fraction of photosynthate to be partitioned to competing plant functions such as the growth of photosynthetic tissue, resource storage and reproduction (Lynch *et al.* 2021b). Contrastingly, the construction of cell walls embedded with more lignin may come at a greater metabolic cost compared to parenchyma cells without the MCS phenotype. Although the carbon costs of the construction of lignin, suberin, cellulose and other cell wall components in root tissue have not been extensively studied, alterations in lignin deposition may be associated with yield penalties (Bonawitz and Chapple 2013; Muro-Villanueva *et al.* 2019). Understanding the metabolic costs of MCS formation, from the perspective of any limiting resource, but most importantly in the context of carbon fluxes and budgets can provide insight into the costs of root elongation or volume of soil explored which is important for deploying this trait in breeding programs and its adaptive value in different environments.

### MCS may influence water and nutrient uptake

Multiseriate cortical sclerenchyma may influence resource acquisition as the root cortex has an important function in apoplastic and symplastic transport of water and nutrients to the vasculature. For example, the deposition of heavily lignified and suberized secondary tissues decreases the absorptive capacity of roots (Rewald *et al.* 2011). In addition, increased cortical width caused by a greater number of cortical cell files and/or an increase in cortical cell size reduces radial conductivity (Heymans *et al.* 2021). Several root anatomical traits including root diameter influence rice and wheat drought tolerance (Kadam *et al.* 2015). Multiseriate cortical sclerenchyma may influence radial water and nutrient uptake through the deposition of heavily lignified tissues and by altering cell-to-cell and apoplastic pathways through smaller cortical cells resulting in a longer path length and therefore influencing the uptake of soil resources. Presumably, MCS reduces radial water and nutrient transport in the root. As MCS formation is absent near the root tips of axial roots and in lateral roots, the spatial pattern of MCS has functional implications for water capture. The spatial and temporal pattern of MCS in different root classes and tissue ages presumably interacts with dynamic water availability in the soil profile to influence root water uptake and plant growth and productivity.

In water-limited environments, a reduced radial conductivity may be advantageous by enabling ‘water banking’, or the conservation of soil water throughout the growth season (He *et al.* 2016; Wang *et al.* 2016). A reduced hydraulic conductivity may be advantageous under water deficit by preventing desiccation of mature roots, the root tip and soil surrounding the root tip. In drought environments, conservation of soil water throughout the growth season contributes to reproductive success and greater yield (Lynch *et al.* 2014). The development of MCS, which presumably influences radial water transport, may be an adaptive trait in water-limiting environments through promoting water banking, but a functionally maladaptive trait in environments with optimal water or nutrient limitation by reducing water and nutrient transport. Nevertheless, MCS forms in axial roots and is absent in lateral roots (Schneider *et al.* 2021). Lateral roots comprise the majority of the root length and are responsible for the majority of nutrient and water capture (Ahmed *et al.* 2015; Schneider and Lynch 2017; Schneider *et al.* 2017a, 2020b). Since MCS does not develop in lateral roots, MCS may not be a benefit or trade-off to overall water or nutrient capture of the plant. After the development of MCS in axial roots, lateral roots may perform the majority of root water and nutrient uptake and the development of MCS may have little to no effect on total resource uptake of the plant, similarly to the root cortical senescence phenotype in barley (Schneider and Lynch 2017; Schneider *et al.* 2020b). The influence of MCS on nutrient and water uptake may be adaptive, maladaptive or neutral for crop fitness and yield depending on the environment and interaction with other plant traits. The influence of MCS on water and nutrient uptake of the plant and its impact on plant fitness is essential to understand in the context of specific environments and interaction with other root traits before integration into crop breeding programs.

Root anatomical phenotypes have not only been shown to impact radial transport, but also radial water loss. Several root anatomical phenotypes that have been shown to reduce radial transport of water also reduce radial water loss. For example, the development of root cortical senescence involves the programmed cell death of cortical cells. Enhanced endodermal suberization was detected after the development of root cortical senescence in barley, which resulted in reduced radial conductivity and radial water loss of the root (Schneider *et al.* 2017b). Presumably, MCS also results in reduced radial water loss through the encrusting of cortical cells with lignin. Multiseriate cortical sclerenchyma could be an important trait in axial roots in water-limiting environments by reducing radial water loss to the rhizosphere and therefore improving plant water status and yield.

Multiseriate cortical sclerenchyma may also influence water and nutrient capture through enabling penetration of hard soils (see section II (Schneider *et al.* 2021)). In compacted soils, roots with MCS were able to grow deeper and subsequently had greater plant performance, presumably due to greater soil resource capture (Schneider *et al.* 2021). However, recently another strategy for plant productivity in compacted soils has been demonstrated. Roots also sense mechanical impedance through ethylene, as soil compaction reduces gas diffusion through a reduction in the number and size of air-filled pores. This accumulation of ethylene at the root tip in compacted soil triggers a suite of hormones that restrict root growth (Jacobsen *et al.* 2021; Pandey *et al.* 2021; Huang *et al.* 2022). Multiseriate cortical sclerenchyma and this ethylene ‘stop signal’ suggest that there may be several strategies of plants to optimize root forage in soils with greater mechanical impedance.

### MCS may influence interactions with edaphic organisms

Root interactions with arthropods and nematodes also have significant impacts on soil resource capture, plant growth and yield. The incorporation of lignin in roots can provide physical and mechanical barriers to herbivory (Meihls *et al.* 2012; Moore and Johnson 2017). Endodermal and exodermal barriers in maize roots that are suberized and lignified act as a barrier to discourage larval feeding of western corn rootworm (Riedell and Kim 1990). Similarly, the cutting resistance of maize roots correlated with the intensity of western corn rootworm feeding on the cortex (Castano-Duque *et al.* 2017) and tobacco roots with greater lignin concentration had delayed penetration by wireworms (Johnson *et al.* 2010). The deposition of secondary metabolites has important roles in determining the potential resistance of the cortex to a number of pests. Presumably, the deposition of lignin during the formation of MCS would also confer resistance to edaphic pests, which would make MCS a desirable breeding target.

The development of highly lignified MCS tissue may also mechanically inhibit colonization of symbionts. For example, roots with sclerenchyma in the outer cortex had decreased arbuscular mycorrhizae colonization (Dreyer *et al.* 2010). However, fine roots (e.g. lateral roots) have increased active arbuscular mycorrhizae colonization compared to lignified, coarse and older roots within the same plant (Guo *et al.* 2008), and the lack of MCS formation in lateral roots may indicate that MCS in axial roots does not significantly influence these interactions. The relationship between anatomical traits and colonization between symbionts is poorly understood and presumably involves many small anatomical and environmental nuances (Strock *et al.* 2019). From a physiological perspective, the relative contribution of the symbiosis compared to the root anatomical trait itself for resource capture or plant growth would reveal the importance of the different mechanisms for plant productivity. It is critical to understand the fitness landscape of MCS and its benefits and trade-offs for pest resistance, symbiotic interactions and subsequently plant fitness and yield in specific environments.

### MCS may influence root exudation and the microbiome

Studies have suggested that root exudation benefits plants through stimulation of beneficial microbes and promoting nutrient acquisition (Mommer *et al.* 2016). How root anatomy, and therefore the biochemical composition and thickness of the root (particularly the outermost cortical layer) influences root exudates along root systems, across different root classes, and in different species remains an open question that has implications for crop productivity and yield. Presumably, outer cortical tissues represent major control points for root exudation and act as a barrier for the release of root exudates that diffuse through the apoplastic spaces when lignified or suberized (Canarini *et al.* 2019). Multiseriate cortical sclerenchyma, influencing the biochemical composition and cell wall thickness of cortical cells, has the potential to influence root exudation and the microbiome by altering the apoplastic space and the length of the symplastic pathways through which exudates travel (Galindo-Castaneda *et al.* 2022). In addition, several root traits including MCS presumably alter oxygen and water content and nutrient availability in the rhizosphere and also influence carbon rhizodeposition to determine the structural and physical characteristics of niches and microhabitats for microbial communities (Galindo-Castaneda *et al.* 2022). It has been proposed that plants with MCS may have reduced exudate secretion and cell shedding resulting in a less diverse and less abundant microbial rhizosphere community. Roots with MCS may also be reduced colonization of endophytes entering the root via the rhizosphere (Galindo-Castaneda *et al.* 2022). However, since MCS is not formed in lateral roots which also contribute to root exudation (Badri and Vivanco 2009), it remains unclear how much a reduction in axial root exudation after MCS formation will influence these interactions and processes. The link between spatiotemporal variation in MCS with other phenotypes including exudates, carbon rhizodeposition and metabolite concentration is poorly understood but crucial for understanding root-microbial associations that have the potential to improve nutrient and water capture and plant productivity (Galindo-Castaneda *et al.* 2022). Optimal combinations of root phenotypes and microbiomes can be matched together to improve plant growth and yield, particularly in environments with stress including suboptimal nitrogen availability and drought (Lynch *et al.* 2021b; Birt *et al.* 2022; Galindo-Castaneda *et al.* 2022).

### MCS may have synergistic interactions with other root traits

While variation for MCS can have significant effects on resource capture and plant growth, the integration of multiple anatomical and architectural traits may also have synergistic or antagonistic effects for resource acquisition and crop performance. Breeding strategies considering the interaction between root traits are especially important in environments where multiple stresses exist and the availability of limiting resources is spatially and temporally variable (Lynch 2021). Understanding the idiosyncratic strategies underlying crop productivity requires investigation of the broader interactions between both above- and below-ground traits that affect soil resource acquisition, transport and utilization.

Interactions between different root traits are important for soil resource capture (Lynch *et al.* 2021b). Several synergisms between root anatomical and architectural traits have been explored for enhanced resource capture (Postma and Lynch 2011; York *et al.* 2013; Schneider *et al.* 2017a, 2020c); however, the identification of synergistic and antagonistic interactions between anatomical traits is lacking. The coupling of fewer cortical cell files with larger cortical cells has been suggested as a synergistic combination to facilitate deeper rooting (Lynch 2013), and plants with thick roots with a larger proportion of stele and smaller distal cortical cells have been proposed as an integrated anatomical phenotype for drought tolerance (Klein *et al.* 2020). Thick roots with a large stele have been reported as an advantageous root phenotype to best surmount mechanical impedance by maximizing root penetrative ability (Colombi and Walter 2016). However, many of these potential interactions between cortical traits remain to be explored and it is likely that root anatomical and architectural traits have many important interactions with each other, with the shoot phenotype, and the environment to influence plant performance and yield.

The potential for the cortex to develop successive and potentially synergistic phenotypes is mostly unknown. For example, barley has the capacity to develop root cortical aerenchyma, root cortical senescence and MCS, among other cortical traits. Nevertheless, it is unknown if barley genotypes have the capacity to develop these root phenotypes successively or simultaneously. For example, whether genotypes could develop MCS early in growth, which subsequently develops into root cortical senescence, remains an open question. If several of these cortical traits could be developed successively, or if genetic variation exists for this phenomenon, these traits could have potentially synergistic effects with utility for plant growth in stress conditions. For example, root cortical aerenchyma (Striker *et al.* 2007) and presumably root cortical senescence decrease the mechanical strength of the root, while MCS enhances root tensile strength (Schneider *et al.* 2021). If these traits are expressed simultaneously or successively, they could have important functional interactions for the mechanical strength of the root and penetration of hard, dry soils and ultimately plant productivity. Plant breeders could select for multiple and potentially successive traits that synergistically interact to improve plant growth and yield.

### MCS may influence soil carbon deposition

The ability of plants to sequester carbon from the atmosphere into deep soil domains is also an important goal for global agriculture (Chabbi *et al.* 2017) and supports building soil health and resilience for enhanced crop productivity (Cooper *et al.* 2021). Biosequestration, or the biologically mediated uptake and conversion of atmospheric CO_2_ into inert, long-lived carbon-containing materials, has the ability to reduce the concentration of atmospheric carbon of important greenhouse gases while creating significant economic land value. Increased soil carbon can be achieved through reducing the rate of decomposition of organic matter in the soil or increasing the rate of carbon additions to the soil.

Tissue composition has been shown to influence the rate and extent of residue decomposition. In general, all plants contain the same classes of organic compounds including cellulose, hemicellulose, starches, proteins, lipids and polyphenols but the proportions of each, which are influenced by species, genotype, maturity and specific plant traits (e.g. MCS), affect the rate of decomposition (Stott *et al.* 1986). Recalcitrant carbon forms, particularly lignin, increase carbon sequestration because they have a long residence time in soil due to the specificity of lignin-degrading enzymes (De Deyn *et al.* 2008). Lignin is a complex polymer of phenylpropane units cross-linked to each other with a variety of chemical bonds and therefore constitutes the most recalcitrant component of the plant cell wall. Subsequently, lignin may also influence the bioavailability of other cell wall components as lignin molecules may physically restrict access to other cell wall components (e.g. cellulose and hemicellulose) and reduce the surface area available to enzymatic penetration and activity. Importantly, the degradation of lignin by soil microbes requires a complex set of carbohydrate-degrading enzymes, which are often not allocated within a single organism. For example, albeit certain white rot fungi, brown rot fungi and soft rot fungi were shown to be capable of completely metabolizing intact lignin molecules (Kirk and Farrell 1987; Bernhard-Reversat and Schwartz 1997); other fungi and bacteria are known to have genes involved just in specific steps of lignin degradation (Crawford and Crawford 1980). The development of MCS, a lignified cortical tissue, may have potential for enhanced soil carbon sequestration through the deposition of recalcitrant forms of carbon (e.g. lignin) in deep soil domains.

## MCS Has Potential in Plant Breeding Programs

Multiseriate cortical sclerenchyma has potential for incorporation into breeding programs for the development of more efficient and productive crop cultivars. Multiseriate cortical sclerenchyma has the potential to substantially influence many important plant functions including water and nutrient uptake and resistance to pests. Genetic variation in MCS has been observed in many important crop species including maize, wheat, sorghum and barley. The heritability of MCS is relatively high (in maize *H*^2^ = 0.64) (Schneider *et al.* 2021), which suggests that this trait could be successfully selected for in breeding programs using either phenotypic selection or marker-assisted selection. In addition, a candidate gene has been identified to be associated with MCS. Genome-wide association analysis and quantitative trait loci mapping in maize revealed MCS to be associated with a genetic marker encoding a MEI2-like RNA-binding protein (Schneider *et al.* 2021). MEI2-like genes are associated with morphogenesis in plants and are required in early formation of the plant (Alvarez 2002). This gene is root-expressed and has elevated expression in root cortical cells and in younger nodes (Stelpflug *et al.* 2015). The confirmation of the role of this candidate gene and identification of additional genes controlling MCS formation will provide molecular tools for breeders to develop improved crop cultivars with enhanced edaphic stress tolerance.

Notably, the formation of MCS in later growth stages during the emergence of roots from younger shoot nodes does not enable phenotypic selection or phenotyping of this trait at the seedling stage or in young plants, which is typically used to accelerate phenotypic selection in breeding programs. In addition, the elevated lignin concentrations in roots with MCS were not correlated with lignin concentration in the stems or leaves of the plant (Schneider *et al.* 2021) which limits selection of this trait based on above-ground phenotypes. However, recent advancements in anatomical imaging, phenotyping and modeling (Strock *et al.* 2019, 2022; Heymans *et al.* 2020, 2021) enable high-throughput phenotyping and exploration of functional implications of anatomical traits of mature field-grown plants (e.g. Schneider *et al.* 2020a) which is required for further research on MCS and for its integration into plant breeding programs.

Also important for breeding programs is the fact that development of MCS is restricted to specific taxa. For example, while MCS is commonly developed in modern maize, barley, wheat and sorghum cultivars, it has not been observed in dicot plants or in several other *Poaceae* including rice (Schneider *et al.* 2021). Because different plant taxa do not express all cortical phenotypes, the relevance of model organisms in determining the function and genetic control of MCS is limited. For example, *Arabidopsis* has a simple and highly regular root cortical structure that often only consists of two cortical layers that may not provide the variation needed to understand the function and variability of many cortical phenotypes. In addition, many model species, including *Arabidopsis* do not form MCS, but instead cortical parenchyma are destroyed through secondary growth processes. *Brachypodium* may offer opportunities to study MCS in model species; however, given technological advancements that enable our ability to understand and manipulate crop genomes, as well as recent technological advances in root anatomical phenotyping of mature field-grown plants, it is more efficient to conduct research directly on crop species rather than rely on model species to maximize direct impact for plant breeding programs.

With the exception of a few notable cases (e.g. Richards *et al.* 1989; Burridge *et al.* 2019), breeders have not directly selected for root traits in breeding programs. Several studies have focused on the identification of suitable genetic markers for root traits to be implemented in marker-assisted selection to enhance crop yield (e.g. Hirel *et al.* 2007; Burton *et al.* 2014; Burridge *et al.* 2017; Kadam *et al.* 2017; Tardieu *et al.* 2017; Schneider *et al.* 2020a, 2022). As noted before, genetic markers and a candidate gene have been identified for MCS (Schneider *et al.* 2021). Nevertheless, few successful cases have demonstrated the integration of this knowledge for the development of new crop cultivars. There are a number of challenges in incorporating root traits into breeding programs including difficulties in incorporating genomics-assisted crop improvement into extant breeding programs and the inability to leverage complex traits to deliver new, profitable cultivars to farmers (Blum 2014; Turner *et al.* 2014; Bernardo 2016; Schneider and Lynch 2020).

**Figure 3.**
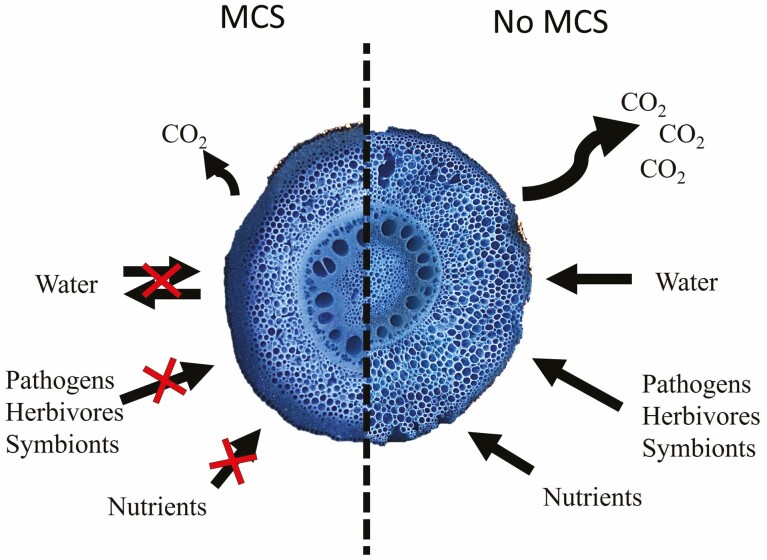
Diagram summarizing the relationships between MCS and soil resource acquisition, metabolic costs and interactions with soil organisms. Multiseriate cortical sclerenchyma is proposed to reduce root respiration, reduce radial water conductivity and radial water loss, improve resistance against pathogens and herbivores, reduce symbiotic associations and reduce radial nutrient transport. Many of these proposed functions of MCS remain to be explored, but have the potential to impact plant health, growth and yield. Images of cross-sections from roots of maize.

Recently, the successful development of new cultivars with root traits for enhanced stress tolerance involving work of crop physiologists and social scientists has laid a framework for the incorporation of root traits into breeding programs (Burridge *et al.* 2019). In addition, the selection of combinations of adaptive alleles or traits for specific environmental conditions through phenomics, modelling and genomic prediction has been proposed as an essential strategy for the development of productive crops (Tardieu 2022). The functional utility of MCS for crop improvement for compaction stress tolerance and the potential of MCS for stress tolerance of additional abiotic and biotic factors and its relatively high make it a strong target for trait-based breeding for the development of more productive crops in edaphic stress (Fig. 3).

## Conclusions

The wide range of genetic variation in MCS within a plant and among and between species suggests that benefits and trade-offs exist. These benefits and trade-offs could involve the ability of the root to develop other, potentially more useful anatomical phenotypes, influence susceptibility to pests and diseases and affect radial nutrient and water transport, microbial associations, among many others. Several important research areas are highlighted to better understand MCS and its potential as a crop breeding target for enhanced stress tolerance. A challenging bottleneck is our incomplete understanding of the fitness landscape of MCS. Understanding the fitness landscape is required to understand the utility of MCS in specific environments, management scenarios and potential interactions with other plant traits. The influence of MCS on plant growth and health is multifaceted over space and time and we must embrace this complexity to better define the fitness optima for specific phenotypes (Lynch *et al.* 2021b; Schneider *et al.* 2021; Schneider 2022). The dearth of previous research on the functional utility of MCS and other anatomical traits combined with new advances in root phenotyping highlights opportunities to study the function and structure of root anatomical traits and offers opportunities for diverse perspectives on these issues. The investigation of broader interactions among multiple cortical, root and plant phenotypes may reveal synergistic relationships among different plant tissues and organs. Multiseriate cortical sclerenchyma merits consideration as a target in plant breeding programs as a trait for plant adaptation to edaphic stress.

## Data Availability

No new data were generated or analysed in support of this research.

## Notes

**Form & Function. Chief Editor:** Kate McCulloh
